# Global Burden of Pancreatic Cancer Among Individuals Aged 15–59 Years in 204 Countries and Territories, 1990–2021: A Systematic Analysis for the GBD 2021 and Projections to 2045

**DOI:** 10.3390/cancers17111757

**Published:** 2025-05-23

**Authors:** Zeyu Xia, Wenping Han, Haigang Niu, Hui Dong

**Affiliations:** 1 People’s Hospital of Yiyang, Yiyang 413001, China; 2 Teaching and Research Section of Surgery, Faculty of Clinical Medicine, Fenyang College, Shanxi Medical University, Fenyang 032200, China; 3 Fenyang Hospital of Shanxi Province, Fenyang 032200, China

**Keywords:** 15–59-year-old cohort, GBD 2021, pancreatic cancer, public health strategies, global, China

## Abstract

Background: Pancreatic cancer (PC), the third leading cause of cancer-related mortality globally, exhibits a persistently low five-year survival rate (13%). While the global burden of PC among individuals aged 15–59 years has declined, trends in China remain understudied. This study evaluates global and national trends in PC incidence, mortality, and disability-adjusted life years (DALYs) from 1990 to 2021 and projects trajectories to 2045. Methods: Using data from the Global Burden of Disease (GBD) 2021 study, we calculated age-standardized rates (ASRs) for 204 countries/territories. Joinpoint (version: 5.3.0.0) regression identified temporal trends via average annual percentage changes (AAPCs), and Bayesian age-period-cohort (BAPC) modeling forecasted future burdens. Results: Globally, PC burden declined among 15–59-year-olds (AAPC for incidence: −0.8%, 95% UI: −1.2 to −0.4). However, China experienced a significant reversal after 2009, with incidence rising by 1.5% annually (95% UI: 0.9–2.1), disproportionately affecting males. Smoking (contributing to 22.2% of DALYs in China) and high fasting plasma glucose (15%) emerged as key modifiable risk factors, while elevated BMI exacerbated burdens in high SDI regions (3.1% of DALYs). Projections indicate a continued surge in China’s PC burden by 2045, particularly among males (incidence projected to increase by 50% from 2010 to 2045). Conclusions: High SDI regions exhibit concentrated PC burdens linked to lifestyle factors, whereas China’s rising trends align with healthcare expansion and metabolic disease proliferation. Targeted interventions—smoking cessation, glycemic control, and weight management—are imperative to mitigate growing burdens in younger populations. This study highlights the urgent need for region-specific strategies to address evolving epidemiological challenges in PC prevention and control.

## Simple Summary

Pancreatic cancer is a leading cause of cancer-related deaths worldwide, with low survival rates. This study examines its impact on people aged 15–59 across 204 countries from 1990 to 2021 and predicts future trends up to 2045. While global rates of pancreatic cancer in this age group have declined, China has seen a rise since 2009, especially among men. Key risk factors include smoking, high blood sugar, and obesity in wealthier regions. The findings highlight that China’s burden is projected to keep increasing, requiring urgent action. Public health efforts should focus on reducing smoking, improving blood sugar control, and managing weight. These strategies could help lower the disease’s impact, particularly in younger populations, and guide governments in allocating resources effectively to save lives and improve health outcomes.

## 1. Introduction

Pancreatic cancer is the third leading cause of cancer death, and the five-year survival rate is only 13% [1]. A substantial proportion of patients exhibit low responsiveness to subsequent chemotherapeutic, radiotherapeutic, and immunotherapeutic interventions [2]. This insensitivity renders the effective management of the disease a formidable challenge. Epidemiological projections indicate that, by 2030, PC is likely to emerge as the second leading cause of cancer-related mortalities [3].

In accordance with the cancer statistics of 2023, an estimated 64,050 new cases of PC and 50,550 related deaths are expected [4]. While the incidence of PC typically peaks among individuals aged 60–80 years, there has been a notable upward trend in the occurrence of PC among adults aged 15–59 years [5]. The 15–59-year-old cohort diagnosed with cancer demonstrates unique biological, epidemiological, and clinical profiles that starkly contrast with those of pediatric and geriatric patients [6,7]. This demographic further interactions with a complex constellation of age-specific challenges. These encompass issues such as fertility preservation, the long-term ramifications of treatment, substantial socioeconomic burdens, and intricate psychosocial aspects [8,9,10]. For patients aged between 15 and 59 years who are diagnosed with cancer, ensuring timely diagnosis, providing optimal care, and implementing targeted treatment strategies are of utmost importance. Consequently, conducting a thorough evaluation of the disease burden and analyzing the epidemiological trends of malignancies within this age group is of critical significance.

The Global Burden of Disease, Injuries, and Risk Factors Study serves as the preeminent global framework for estimating disease burden. It furnishes critical metrics, including age-standardized cancer mortality, incidence, and disability-adjusted life-years (DALYs) [11].

The present study endeavors to comprehensively evaluate the global burden and temporal trends of PC within the 15–59-year-old population. Utilizing the latest data from the 2021 Global Burden of Disease (GBD) studies [12,13], this research will identify significant trends and their driving factors, providing valuable insights for public health planning. Particular emphasis is placed on discerning the patterns of incidence and mortality, as well as unearthing the socioeconomic disparities associated with gender, geographical location, and the level of human development. By highlighting these disparities, the findings of this study aim to inform and underpin targeted cancer control strategies. These strategies are designed to rectify health inequalities and optimize the allocation of resources for this vulnerable subgroup, thereby enhancing the overall quality of cancer care and prognosis within this demographic.

## 2. Materials and Methods

### 2.1. Data Source

The data utilized in this study were sourced from the 2021 Global Burden of Disease (GBD) Study, which has earned international acclaim for its comprehensive and systematic evaluations of disease burdens across diverse regions and countries [12,13]. Specifically, for the present research, data spanning from 1990 to 2021 were retrieved, with a primary focus on pancreatic cancer, both globally and in China. The key metrics of interest encompassed incidence rates, prevalence, mortality numbers, and DALYs. This precisely targeted data extraction strategy enabled a detailed and in-depth examination of the disease burden associated with pancreatic cancer on a global scale and within the Chinese context during this specific temporal period.

### 2.2. Study Design and Population

The study focused on the 15–59-year-old populations in both the global context and China. These populations were stratified according to gender, with the aim of analyzing the trends of pancreatic cancer over a span of three decades. To account for variations in age distribution over time and among different populations, age-standardized rates were computed. This approach ensures that the trends observed accurately mirror the actual changes in the disease burden, rather than being confounded by demographic alterations.

### 2.3. Statistical Analysis

Descriptive statistical methods were meticulously applied to comprehensively summarize crucial parameters associated with pancreatic cancer. These parameters encompass the annual number of cases, prevalence, incidence rates, mortality rates, and disability-adjusted life years (DALYs). To account for potential gender disparities, these metrics were reported separately for males and females. Moreover, age-specific rates were calculated with precision to discern the trends within different age groups, which could provide valuable insights into the disease patterns specific to each age cohort.

Joinpoint regression analysis was rigorously implemented to identify significant alterations in trends over the course of the study period. This analytical approach is capable of detecting the points at which there is a statistically significant change in the linear slope of the trend, as documented in reference [14]. By conducting this analysis, we were able to accurately pinpoint the periods during which there were notable increases or decreases in the burden of pancreatic cancer. This, in turn, offers invaluable insights into the effectiveness of public health interventions and the variations in the prevalence of risk factors, thereby facilitating a deeper understanding of the disease dynamics.

Age-period-cohort (APC) analysis was systematically carried out to disentangle the individual effects of age, period, and cohort on the incidence and mortality rates of pancreatic cancer. The Bayesian APC (BAPC) model was employed to project future trends up to the year 2050. This model enables the simultaneous estimation of age, period, and cohort effects, providing a comprehensive and detailed understanding of how these factors interact and shape the disease trends over time, as described in reference [15]. The APC analysis, therefore, plays a pivotal role in understanding the underlying demographic and temporal factors that drive the observed changes in the incidence and mortality of pancreatic cancer, contributing significantly to the overall understanding of the disease epidemiology.

Using aggregated GBD data can mask regional differences within large countries like China, and therefore there may be reporting bias in cancer registries for certain low SDI countries.

### 2.4. Data Visualization

Data visualization was carried out with R software (version 4.4.2) to create figures showing pancreatic cancer burden trends. Graphs included age-specific incidence, prevalence, and mortality rates, plus joinpoint and decomposition analysis results. These visualizations were crucial for highlighting key findings and trends, making complex data more accessible. Additionally, we used several R packages for data reading, plotting, and analysis. The easyGBDR (version 2.1.7.1) package was used for data reading and visualization, while ggplot2 (version 3.5.2) and ggraph (version 2.2.1) were employed for plotting.

### 2.5. Ethical Considerations

This study utilized publicly accessible data from the GBD database, eliminating the need for ethical approval. All methods were implemented in compliance with relevant guidelines and regulations.

## 3. Results

### 3.1. Disease Burden Due to Pancreatic Cancer by Regions and Countries

**Figure 1:**
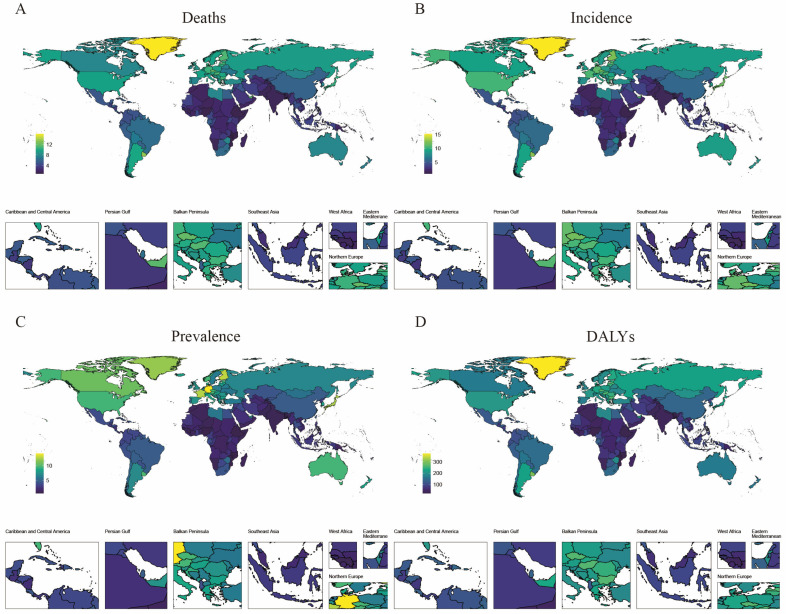
Global distribution maps of the disease burden of pancreatic cancer in 204 countries and regions in 2021. (**A**) Age-standardized death rate (ASDR) in 2021; (**B**) Age-standardized incidence rate (ASIR) in 2021; (**C**) Age-standardized prevalence rate (ASPR) in 2021; (**D**) Age-standardized disability-adjusted life years (DALYs) rate in 2021. ASR stands for age-standardized rate; DALYs stands for disability-adjusted life years.

Figure 1 demonstrates substantial geographical disparities in the 2021 global burden of pancreatic cancer across four epidemiological metrics: incidence, prevalence, disability-adjusted life years (DALYs), and mortality. Regions depicted with warmer hues on the geographic heatmap (e.g., Greenland, Bulgaria, Latvia)—corresponding to higher metric values—demonstrate significantly elevated age-standardized mortality and DALY rates (Figure 1A,D). Greenland presents the highest age-standardized mortality rate (6.16 per 100,000; 95% UI: 8.34–4.36) (Figure 1A), while Western European nations like Germany and France show elevated age-standardized prevalence (Germany: 14.11 [15.17–12.98]; France: 12.54 [13.87–11.42]) (Figure 1C). Notably, China and Russia demonstrate high absolute disease burdens despite lower age-standardized rates.

In contrast, Mozambique displays the world’s lowest pancreatic cancer burden, in relation to age-standardized prevalence (0.71 [0.98–0.54]), mortality (0.90 [1.17–0.68]), and DALYs (19.30 years) (Figure 1D; Table 1). Table 1 further quantifies the disease burden in the top five affected countries/regions, revealing Greenland’s exceptional mortality (15.89 [19.30–12.86]) and DALY rates (374.93 [455.02–306.07]).

### 3.2. Incidence, Prevalence and Mortality of Pancreatic Cancer in China, 2021

**Figure 2:**
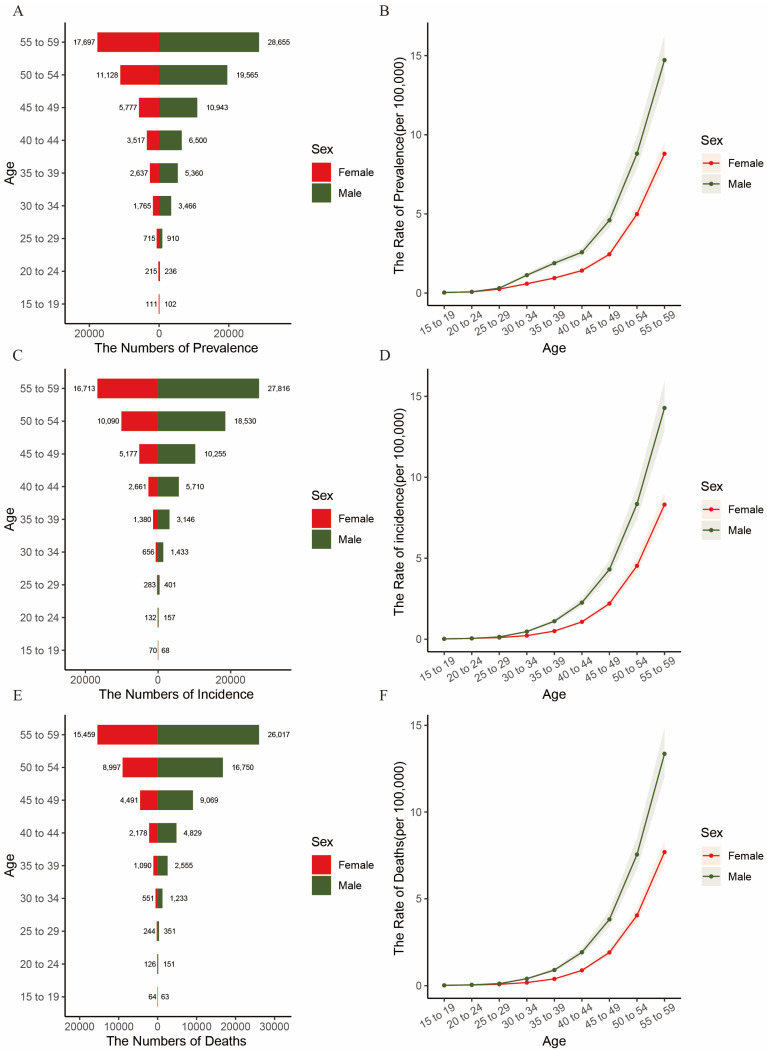
Pancreatic cancer prevalence, incidence, and mortality among 15–59-year-olds globally, by age and gender. In (**A**,**C**,**E**), bar charts show counts for each age group (red for females, green for males, values labeled above). In (**B**,**D**,**F**), curves show rate trends (red for females, green for males); Women are shown on the left.

**Figure 3:**
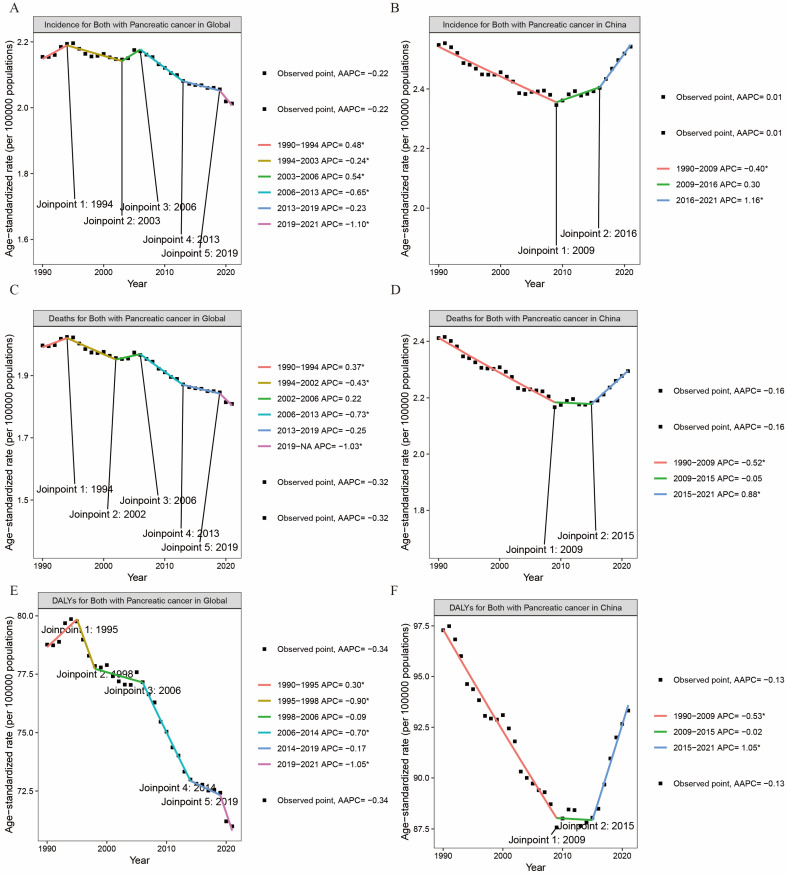
APC and AAPC of ASR for incidence (**A**,**B**), DALYs (**C**,**D**) and deaths (**E**,**F**) in PC at the global and China levels based on the joinpoint regression analysis model (* *p* < 0.05).

In the 2021 global analysis chart of pancreatic cancer disease burden by age and gender, as depicted in Figure 2, it can be observed that, in 2021, among the global population aged between 15 and 59, both the death toll, the incidence, and the prevalence among men and women exhibit an upward trend with increasing age, with the 55–59 age group having the largest number of people. The same trend is also evident in Figure 3. However, it should be noted that the age-standardized prevalence rate, age-standardized incidence rate, and age-standardized mortality rates of men are significantly higher than those of women. In 2021, the number of pancreatic cancer deaths among the global population aged 55–59 was 26,017 for men and 15,459 for women(Figure 2E). Meanwhile, the corresponding data for the Chinese population are 8083 and 4195(Figure S4). The age-standardized mortality rate for men aged 55–59 is 13.36082486 per 100,000, whereas that for women is 7.69103061 per 100,000, reaching the peak value within the 15–59 age group.

### 3.3. Analysis of Trends by Gender Based on the Joinpoint Regression Model, 1992–2021

Figure 3 illustrates the trend of the disease burden related to pancreatic cancer from 1990 to 2021, using the joinpoint regression model. In this figure, the upward trends in the disease burden (manifested as incidence, DALYs, and deaths) for both males and females over the past three decades are presented. It can be seen from Figure 3A,C,D that, globally, the disease burden of pancreatic cancer has witnessed a steady decline among people aged 15–59 years between 1990 and 2021. However, in China, the disease burden of pancreatic cancer has been rising since 2009 (as shown in Figure 3B,D,E), particularly with regard to its incidence. Moreover, the burden of pancreatic cancer in terms of death and disability among people aged 15–59 in China maintained a stable trend from 2009 to 2015, but after 2015, it exhibited a significant upward trend (as depicted in Figure 3D,E).

### 3.4. Cross-Country Social Inequalities Analysis

**Figure 4:**
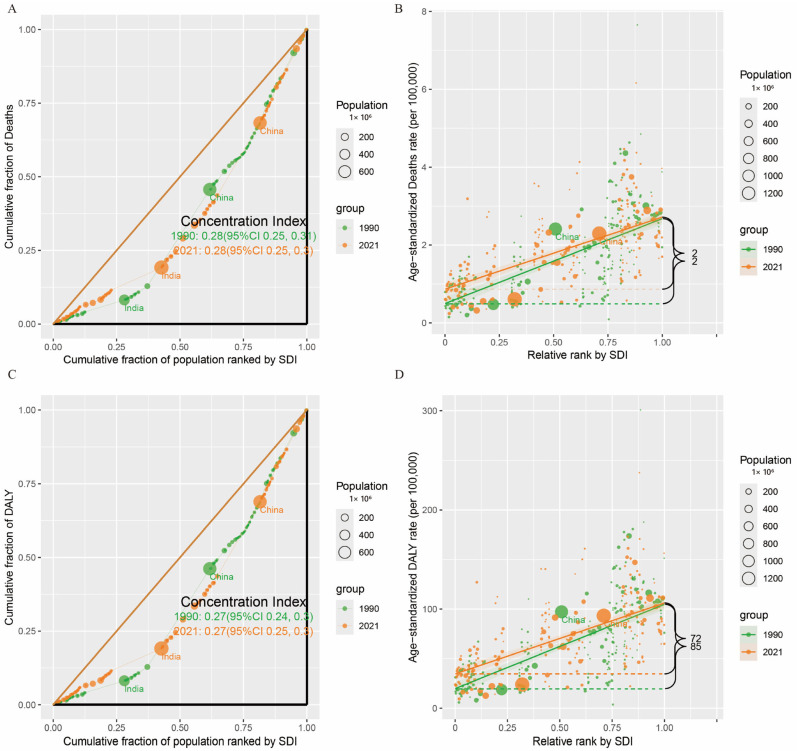
(**A**) Slope index of the ASR-deaths; (**B**) Concentration index of ASR-deaths. (**C**) Slope index of ASR-DALYs; (**D**) Concentration index of the ASR-DALYs.

Using the Slope Index of Inequality (SII) and Concentration Index, absolute and relative inequalities related to SDI were observed in ASDALYs, and ASDR for 15–59-year-old population of pancreatic cancer. In Figure 4A,C, the curves ascend from the lower-left to the upper-right quadrant and lie beneath the fitted line. Coupled with a positive concentration index of 0.28, this indicates that both the cumulative fraction of pancreatic cancer-related deaths (as depicted in Figure 4A) and the number of disability-adjusted life years (DALYs) (in Figure 4C) exhibit a tendency to concentrate in areas with a higher socio-demographic index (SDI). Specifically, as the cumulative proportion of the population ranked according to SDI increases (progressing from left to right along the axis), the cumulative proportion of deaths (in Figure 4A) and that of DALYs (in Figure 4C) concurrently rise. This suggests that, in areas with a relatively elevated SDI, the impact of pancreatic cancer in terms of both mortality and disease burden is more pronounced. In Figure 4B,D, with the variation in the relative rank of SDI, the age-standardized mortality rate of pancreatic cancer (shown in Figure 4B) and the DALY rate (in Figure 4D) demonstrate an upward trend in tandem with the increase in SDI. This further underscores that, across regions or populations at diverse SDI levels, the mortality rate and disease burden rate of pancreatic cancer are relatively higher in regions with a relatively higher SDI. Notably, in Figure 4D, it becomes evident that, in regions with a relatively more favorable economic situation, the DALY rate among the 15–59-year-old population is between 72 (in 2021)–85 (in 1990) per 100,000 individuals, and higher compared to regions with a relatively weaker economic standing.

### 3.5. Predictions of Disease Burden

**Figure 5:**
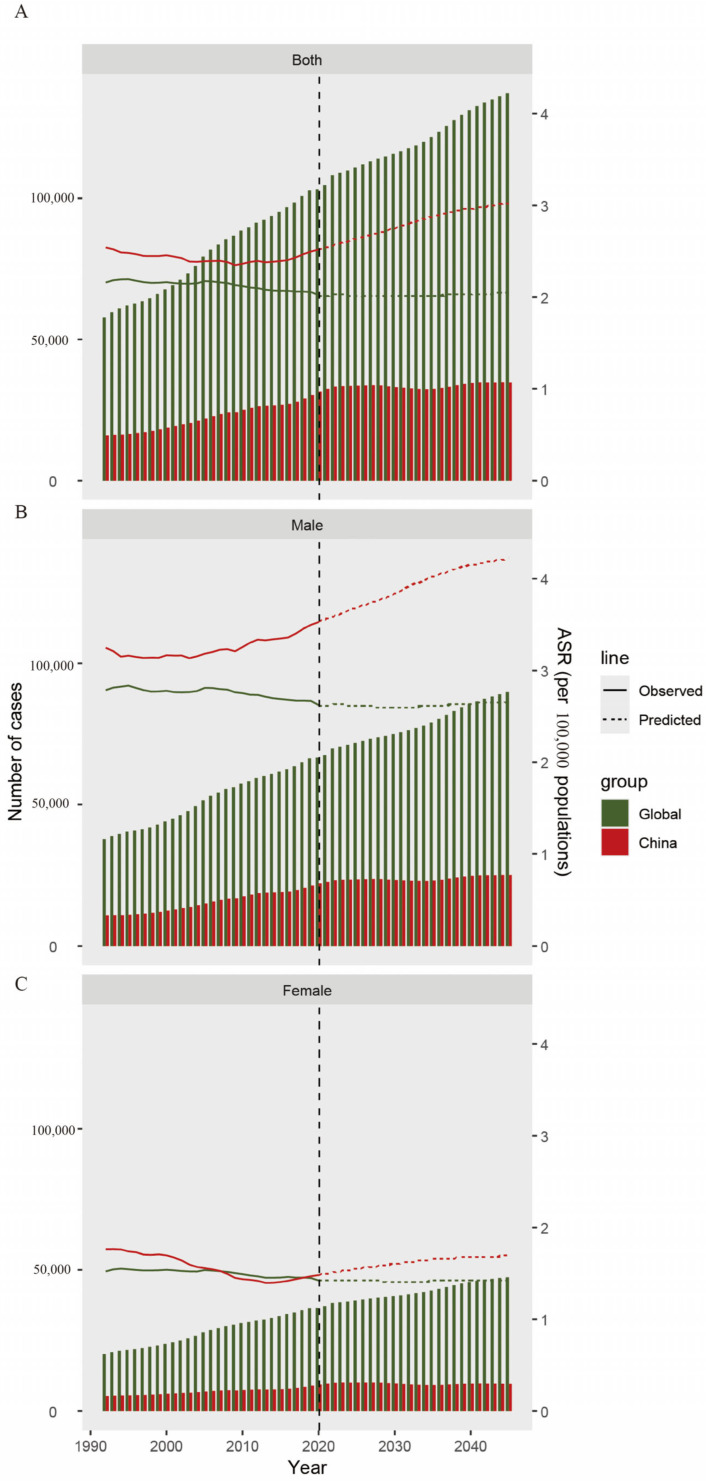
BAPC analysis predicting the incidence of pancreatic cancer in the global and Chinese population aged 15–59 years by 2045. (**A**) BAPC model for incidence in both sexes. (**B**) BAPC model for incidence in males. (**C**) BAPC model for incidence in females.

Figure 5 presents actual and projected data on the burden of disease associated with pancreatic cancer, including ASIRs distributed by sex from 1990 to 2050. Overall, the disease burden of pancreatic cancer among the 15–59-year-old population in China is generally higher than the global level. Additionally, in China, the disease burden of pancreatic cancer among men is higher than that among women. Globally, the age-standardized incidence rates (ASIR) of pancreatic cancer show an overall downward trend (Figure 5A). However, in China, starting from 2010, the disease burden of pancreatic cancer among the 15–59-year-old population has been rising, and it is projected to keep climbing from 2022 to 2050. The increasing trend of incidence data of pancreatic cancer patients in the 15–59-year-old male group in China is more remarkable: from approximately 50,000 people in 2010, it is expected to increase to 75,000 people by 2045 (Figure 5B). Whether at the global level or in China, the disease burden of pancreatic cancer among the 15–59-year-old female group is significantly lower than that among men. The number of female pancreatic cancer patients who die is approximately one-third of that of men (Figure 5C). Supplementary Figures S1 and S2 show ASDALYs, and age-standardized death rate (ASDR)-related disease burden plots.

### 3.6. Attribution Analysis

Due to data limitations in the GBD database, we only obtained pancreatic cancer disease attribution data for the 20–54 age group. This chart presents the disease attribution analysis for pancreatic cancer in the 20–54 age group, focusing on the contribution percentages of three risk factors (high BMI, high fasting plasma glucose, and smoking) to DALYs (disability-adjusted life years) and deaths across different SDI (socio-demographic index) levels and regions. The key results is as follows: Smoking is the primary risk factor. Globally, smoking accounts for 18.2% of DALYs and 18.5% of deaths. In high SDI regions, smoking contributes to 18.6% of DALYs and 18.8% of deaths. China is particularly notable in this respect: smoking accounts for 22.2% of DALYs and 22.5% of deaths, significantly exceeding the global average.

**Figure 6:**
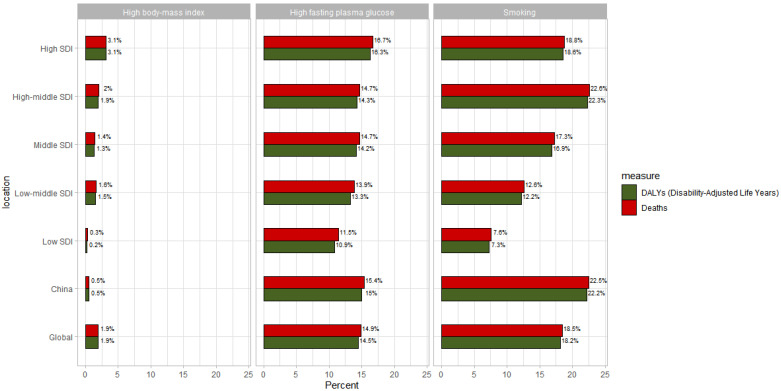
Disease attribution analysis of pancreatic cancer in the 20–54 age group, focusing on the contribution percentages of three risk factors (high BMI, high fasting plasma glucose, and smoking) to DALYs (disability-adjusted life years) and deaths across different SDI (socio-demographic index) levels and regions.

High fasting plasma glucose is the second major risk factor. Globally, it accounts for 14.5% of DALYs and 14.9% of deaths, with little variation across SDI levels. China’s high fasting plasma glucose contribution rate (15%) is close to the global average. High BMI has a relatively lower impact: Globally, it accounts for 1.9% of both DALYs and deaths. In high SDI regions, the contribution rate of high BMI increases significantly (3.1%) in Figure 6.

## 4. Discussion

From the global distribution map of the disease burden of pancreatic cancer across all age groups in 2021, it is evident that Greenland, along with countries and regions on the European continent, bears a relatively heavy disease burden of pancreatic cancer. In contrast, African countries exhibit a notably lighter burden. China’s disease burden of pancreatic cancer is at a moderate level globally. Scientific research has revealed that, since the onset of the 20th century, the Inuit in Greenland have undergone social Westernization. The decline of the traditional hunting lifestyle has led to a reduction in physical activity, accompanied by lifestyle alterations such as smoking and unhealthy dietary habits, which potentially elevate the risk of pancreatic cancer [16]. Regarding the relatively light disease burden of pancreatic cancer in African countries in 2021, this phenomenon may be associated with factors such as the region’s economic level and local dietary customs. Studies indicate that central and eastern sub-Saharan Africa have the lowest incidence of early-onset pancreatic cancer. For instance, Ethiopia reports an incidence of merely 0.14 cases per 100,000 individuals, significantly lower than that in other regions [17]. Additionally, China’s age-standardized incidence rate of pancreatic cancer is approximately one-third of the global average. Research shows that, compared to Western regions, East Asia shoulders a higher burden of gastric, liver, esophageal, and gallbladder cancers, while the burdens of colorectal cancer and pancreatic cancer are increasing [18,19]. As a major East Asian country, China must pay closer attention to the economic, social, and other pressures stemming from the disease burden of pancreatic cancer.

Subsequently, we investigated the distribution of the association between the disease burden of pancreatic cancer and gender among the global and Chinese populations aged 15–59. In 2021, we observed that the age-standardized prevalence, incidence, and mortality rates of pancreatic cancer among males aged 15–59 worldwide were significantly higher than those among females. The 55–59 age group had the highest number of cases. This finding aligns with the research of other scientists [16,20,21,22], possibly attributable to male-specific unhealthy lifestyle habits such as smoking [22], alcohol consumption, and irregular daily routines. Notably, a history of excessive alcohol consumption increases the risk of pancreatic cancer by 2.5 times [23]. Moreover, the rising incidence of pancreatic cancer among individuals aged 15–59 has been linked to an increase in the BMI index, suggesting that obesity may be a contributing risk factor [24,25]. This highlights the need for governments worldwide to prioritize health education on healthy lifestyle habits for men when formulating regional disease prevention and control strategies. Men should also be reminded to undergo regular abdominal ultrasound and CT scans to screen for pancreatic-related diseases.

This study employed a joinpoint regression model to elucidate the trends in the disease burden associated with pancreatic cancer among individuals aged 15–59 from 1990 to 2021. Globally, the disease burden of pancreatic cancer in this age group has been steadily decreasing. However, in China, the disease burden of pancreatic cancer has been rising since 2009, particularly in terms of its incidence rate. Our research team traced the development and found that, in 2009, China initiated a significant medical reform, pledging to provide all citizens with equal access to basic medical care of reasonable quality and financial risk protection. Subsequently, the government’s health funding quadrupled [26]. This change in the healthcare landscape may have enabled earlier detection and treatment of pancreatic cancer. Additionally, as a major East Asian country, several studies conducted between 2019 and 2021 have revealed an upward trend in the incidence of gastrointestinal malignancies, including early-onset colorectal, pancreatic, and gallbladder cancers, in East Asia [22,27,28]. In addition to the advancements in healthcare, the improvement in living standards has also contributed to an increase in metabolic diseases, which in turn may drive the development of pancreatic cancer. A meta-analysis involving 140,000 individuals found that, from 2014 to 2020, the prevalence and incidence of metabolic syndrome increased more rapidly among the young population aged 15 to 40 (from 7.6% to 16.5%) compared to those aged 40 and above (from 33.0% to 35.2%) [29]. Therefore, these research findings may offer valuable insights for the world, countries, and regions to formulate effective prevention and control strategies for pancreatic cancer and reduce its disease burden.

Furthermore, this study analyzed transnational social inequality. We discovered a positive correlation between the disease burden of pancreatic cancer among individuals aged 15–59 and the socio-demographic index (SDI) classification. That is, in more economically developed regions, the disease burden of pancreatic cancer is relatively higher, and this pattern has remained largely unchanged from 1992 to 2021 (CI = 0.27). This result is consistent with previous findings [30,31], highlighting the significant disparities in the burden of pancreatic cancer across different regions. High SDI regions typically exhibit the highest incidence and mortality rates, while lower SDI regions show a downward trend. This may be attributed to factors such as a higher proportion of an aging population, unhealthy lifestyle habits, and metabolic disorders in high SDI countries. The higher incidence rates observed in high SDI regions may also be associated with greater access to imaging technology and enhanced health awareness. In regions with a relatively high SDI, people are more health-conscious and have better access to radiological services [32], enabling the detection of more incidental cancers, even before the onset of symptoms.

The study examined the actual and predicted data on the disease burden related to pancreatic cancer. We found that the disease burden of pancreatic cancer among individuals aged 15–59 has been increasing and is projected to continue rising from 2022 to 2050. Notably, the upward trend in the incidence of pancreatic cancer among Chinese males aged 15–59 is particularly significant. This finding underscores the need to enhance the prevention and treatment of pancreatic cancer in this population globally, especially in China (Figure S3). It also provides valuable insights for the formulation of major public health policies and the promotion of health education.

Tobacco use and elevated fasting plasma glucose remain the primary modifiable risk factors for pancreatic cancer burden in individuals aged 20–54 years, particularly within medium- and high SDI regions. While global tobacco control initiatives have successfully reduced smoking prevalence through coordinated policy measures [33,34], our analysis demonstrates that smoking continues to contribute substantially to pancreatic cancer pathogenesis, likely through chronic exposure to tobacco-specific carcinogens that promote cellular mutagenesis. Concurrently, sustained hyperglycemia exhibits comparable epidemiological significance, where pancreatic β-cell dysfunction and insulin resistance may foster a pro-oncogenic microenvironment via chronic inflammatory pathways. These findings necessitate dual preventive strategies: intensified tobacco control policies—particularly in China through legislative enforcement—and systematic metabolic monitoring for diabetic populations, including regular abdominal imaging surveillance. Public health priorities should further emphasize weight management and investigation of undercharacterized risk factors in low SDI regions, while maintaining methodological rigor in risk attribution analyses through standardized Global Burden of Disease frameworks.

In this study, we constructed the APC, BAPC, and joinpoint models to analyze the disease burden of pancreatic cancer among those aged 15 to 59 globally and in China, obtaining the above results. However, the BAPC model and joinpoint regression analysis are based on assumptions that may oversimplify cancer trends. They assume that the impacts of age, period, and cohort on cancer incidence and mortality are additive and independent, failing to fully capture the complex interactions among these factors. Also, these models assume smooth and continuous changes in cancer trends, potentially missing abrupt changes caused by external factors like policy shifts, environmental influences, or medical breakthroughs. In addition, this study has limitations. The data from the Global Burden of Disease (GBD) study may vary in accuracy and completeness, possibly affecting the reliability of our findings [35]. Moreover, the disparities in healthcare accessibility and quality across different regions in China have not been comprehensively studied, which may influence early detection and treatment outcomes.

## 5. Conclusions

In 2021, the disease burden of pancreatic cancer remained formidable. From 1990 to 2021, the disease burden of pancreatic cancer among individuals aged 15–59 exhibited a downward trend globally. However, in China, since 2009, the age-standardized incidence rate, mortality rate, and disability-adjusted life years have all been rising. From the perspective of gender and age, males, particularly those in the 55–59 age group, bear a heavier disease burden of pancreatic cancer. Through BAPC, joinpoint analysis, concentration index analysis, and slope index analysis, we found that, from 1992 to 2021, the disease burden of pancreatic cancer among individuals aged 15–59 was higher in high SDI regions and relatively lower in low SDI regions. Disease burden predictions indicate that the disease burden of pancreatic cancer among individuals aged 15–59 in China is expected to continue increasing from 2022 to 2050. The burden of pancreatic cancer is associated with smoking and high fasting plasma glucose. Additionally, high BMI in populations from high SDI regions also contributes significantly to the disease burden. This research holds significant reference value for the prevention, treatment, and health education of pancreatic cancer among individuals aged 15–59 globally and, in particular, in China.

## Figures and Tables

**Table 1:** ASDR, ASPR, ASIR and age-standardized disability-adjusted life years (DALYs) rates of the top five countries in 2021.

Measure	Location	Val	Upper	Lower
**Deaths**	Greenland	6.163709	8.339102	4.3568361
	Bulgaria	4.465164	5.432986	3.6239335
	Latvia	4.372814	5.401602	3.5037153
	Romania	4.175437	5.054531	3.3858231
	Uruguay	4.134278	4.856886	3.4793197
**Incidence**	Greenland	6.653686	8.981315	4.7168994
	Latvia	4.799047	5.927480	3.8498763
	Bulgaria	4.786269	5.831670	3.8642011
	Monaco	4.562883	7.189736	2.6233051
	Uruguay	4.491356	5.278130	3.7705617
**DALYs**	Greenland	237.61867	322.12296	167.39080
	Bulgaria	173.03564	210.83126	140.01923
	Latvia	170.78119	211.53262	136.72399
	Romania	161.27127	195.47092	130.79996
	Uruguay	160.94532	189.21098	135.16508
**Prevalence**	France	7.838120	9.508800	6.3849828
	Germany	7.675885	9.226489	6.3230564
	Greenland	6.928199	9.393575	4.8729118
	Monaco	5.832303	9.342838	3.249271
	Latvia	5.037147	6.210564	4.041666

## Data Availability

The datasets used during the current study are available from the corresponding author upon reasonable request.

## Supplementary Materials

The following supporting information can be downloaded at: https://www.mdpi.com/article/10.3390/cancers17111757/s1, Figure S1: BAPC analysis predicting the deaths of pancreatic cancer in the global and Chinese population aged 15–59 years by 2045. (A) BAPC model for deaths in both sexes. (B) BAPC model for deaths in males. (C) BAPC model for deaths in females. Figure S2: BAPC analysis predicting the DALYs of pancreatic cancer in the global and Chinese population aged 15–59 years by 2045. (A) BAPC model for DALYs in both sexes. (B) BAPC model for DALYs in males. (C) BAPC model for DALYs in females. Figure S3: Age, Period, and Birth Cohort Effects on 15–59-year-old Pancreatic Cancer Mortality. (I): Global; (II): China. (A): Age Cohort; (B): Period Cohort; (C): Birth Cohort. Figure S4: Pancreatic cancer prevalence, incidence, and mortality among 15–59-year-olds in China, by age and gender. In (A,C,E), bar charts show counts for each age group (red for females, green for males, values labeled above). In (B,D,F), curves show rate trends (red for females, green for males); Women are shown on the left.

## Abbreviations

DALYs	Disability-adjusted life-years
ASDR	Age-standardized death rate
ASIR	Age-standardized incidence rate
ASPR	Age-standardized prevalence rate
PC	Pancreatic cancer
APC	Age-period-cohort
ASR	Age-standardized rate
