# Supplementary material for: Global Burden of Pancreatic Cancer Among Individuals Aged 15–59 Years in 204 Countries and Territories, 1990–2021: A Systematic Analysis for the GBD 2021 and Projections to 2045
Source: Cancers (Basel). 2025 May 23;17(11):1757. doi: 10.3390/cancers17111757 (PMC12153570; doi:10.3390/cancers17111757)
Supplement: Supplementary file 1 [file cancers-17-01757-s001.zip › cancers-3595966-supplementary.pdf]

Supplementary data

1. **Predictions of disease burden**

Figure S-1 presents actual and projected data on the burden of disease associated with pancreatic cancer, including ASDRs distributed by sex from 1990 to 2045.Figure S-2 presents actual and projected data on the burden of disease associated with pancreatic cancer, including DLAYs ASR distributed by sex from 1990 to 2045.

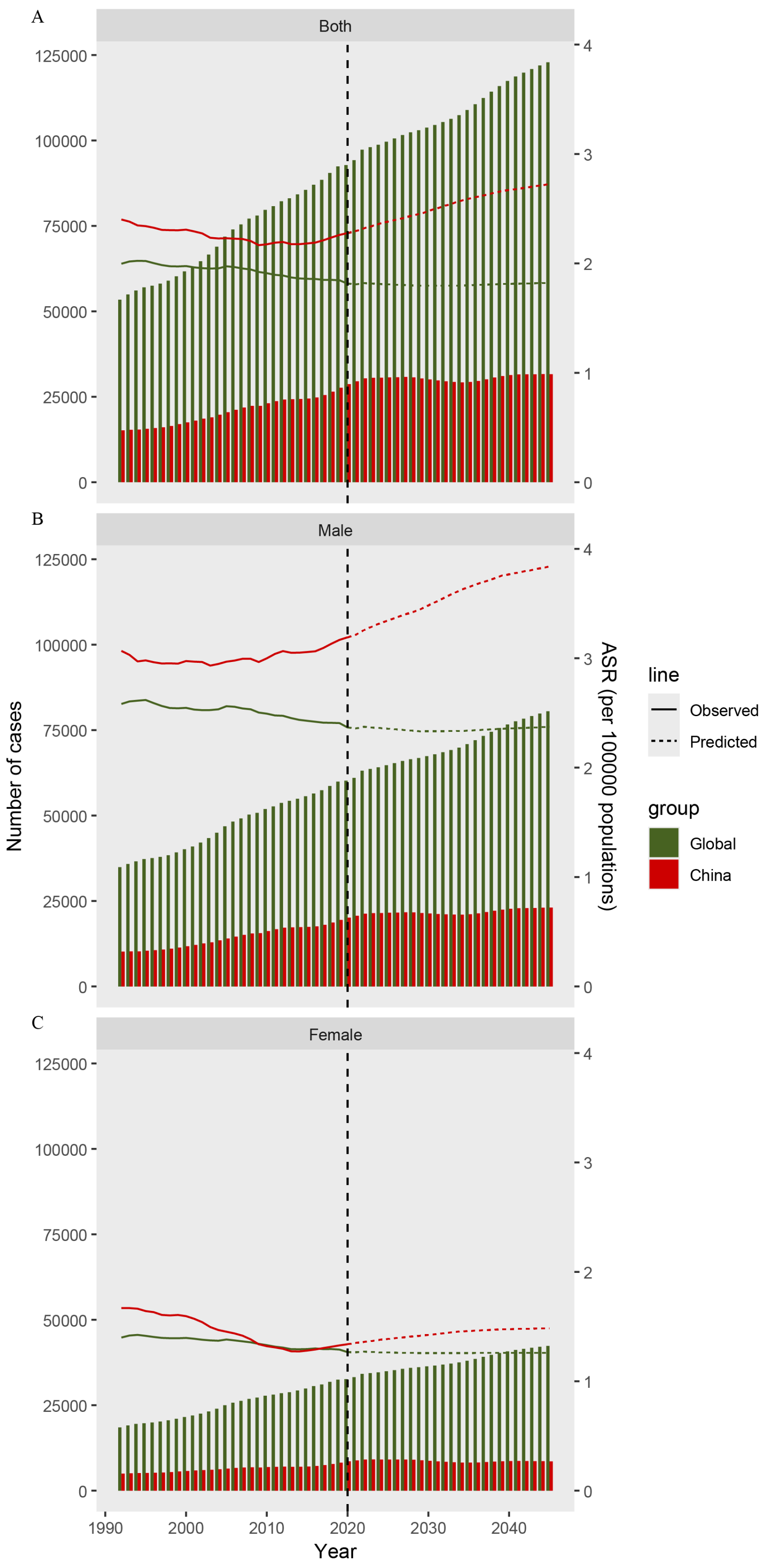

Figure S-1: BAPC analysis predicting the deaths of pancreatic cancer in the global and Chinese population aged 15–59 years by 2045. (A) BAPC model for deaths in both sexes. (B) BAPC model for deaths in males. (C) BAPC

model for deaths in females.

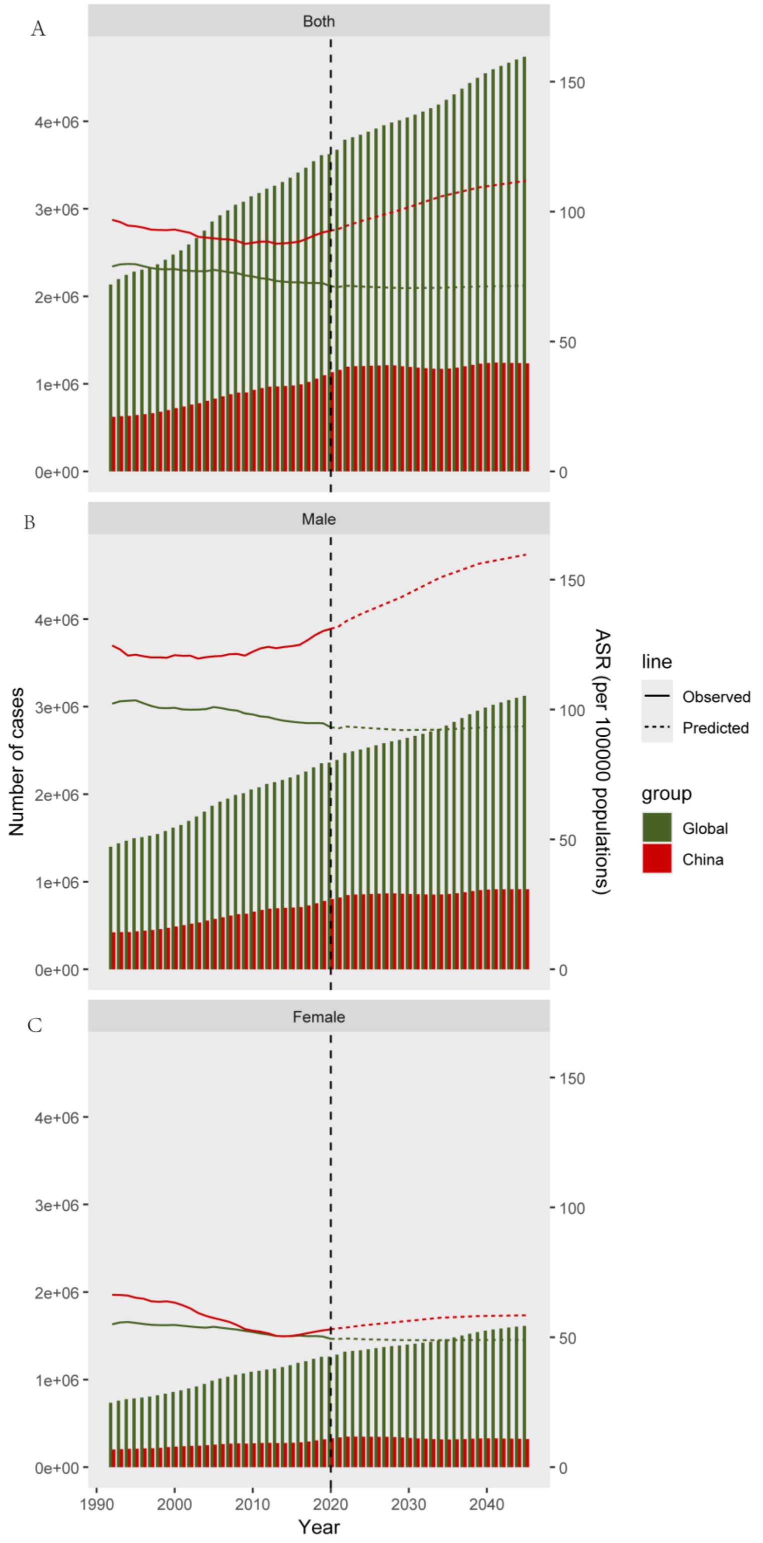

Figure S-2: BAPC analysis predicting the DALYs of pancreatic cancer in the global and Chinese population aged 15﹣59 years by 2045. (A) BAPC model for DALYs in both sexes. (B) BAPC model for DALYs in males. (C) BAPC model for DALYs in females.

The impacts associated with age, period, and birth cohort on pancreatic cancer mortality

The age, period, and birth cohort (APC) model was employed to comprehensively analyze the age - related, period - related, and birth cohort - related effects on pancreatic cancer mortality among individuals aged 15 - 59 years. The findings, as presented in Supplementary Figure 3, revealed distinct trends at both the global and Chinese levels. Globally, the overall mortality rate of pancreatic cancer among the 15 - 59 age group has exhibited a dynamic downward trajectory. This is clearly evidenced by the consistently negative annual change values, as shown in Supplementary Figure 3 - IA. The period effect analysis further corroborated this decline, with all relative risk (RR) values being less than 1. This indicates that, when compared to the reference period, the relative risk of pancreatic cancer - related death during the study period was significantly lower, as depicted in Supplementary Figure 3 - IB. Additionally, the birth cohort analysis demonstrated that for individuals born since 1976, there has been a notable decrease in pancreatic cancer - related mortality, with RR values consistently below 1 (Supplementary Figure 3 - IC). In contrast, the situation in China presents a different picture. The mortality rate of pancreatic cancer among the 15 - 59 age group in China has remained relatively stable. Particularly, among Chinese men, the annual change rate was positive, with the trend line hovering around the zero - value mark, as shown in Supplementary Figure 3 - IIA. Alarmingly, when examining the period effect, the RR values for Chinese men have been on an upward trend since 1992 (Supplementary Figure 3 - IIB). In the birth cohort analysis, while the global trend shows a decline in pancreatic cancer mortality for those born since 1976, Chinese men have contributed to an increase in the number of pancreatic cancer - related deaths in China during this period, with RR values consistently greater than 1 (Supplementary Figure 3 - IIC). These findings underscore the need for targeted public health interventions and research efforts, especially in China, to address the rising risk of pancreatic cancer mortality among men in the 15 - 59 age group.

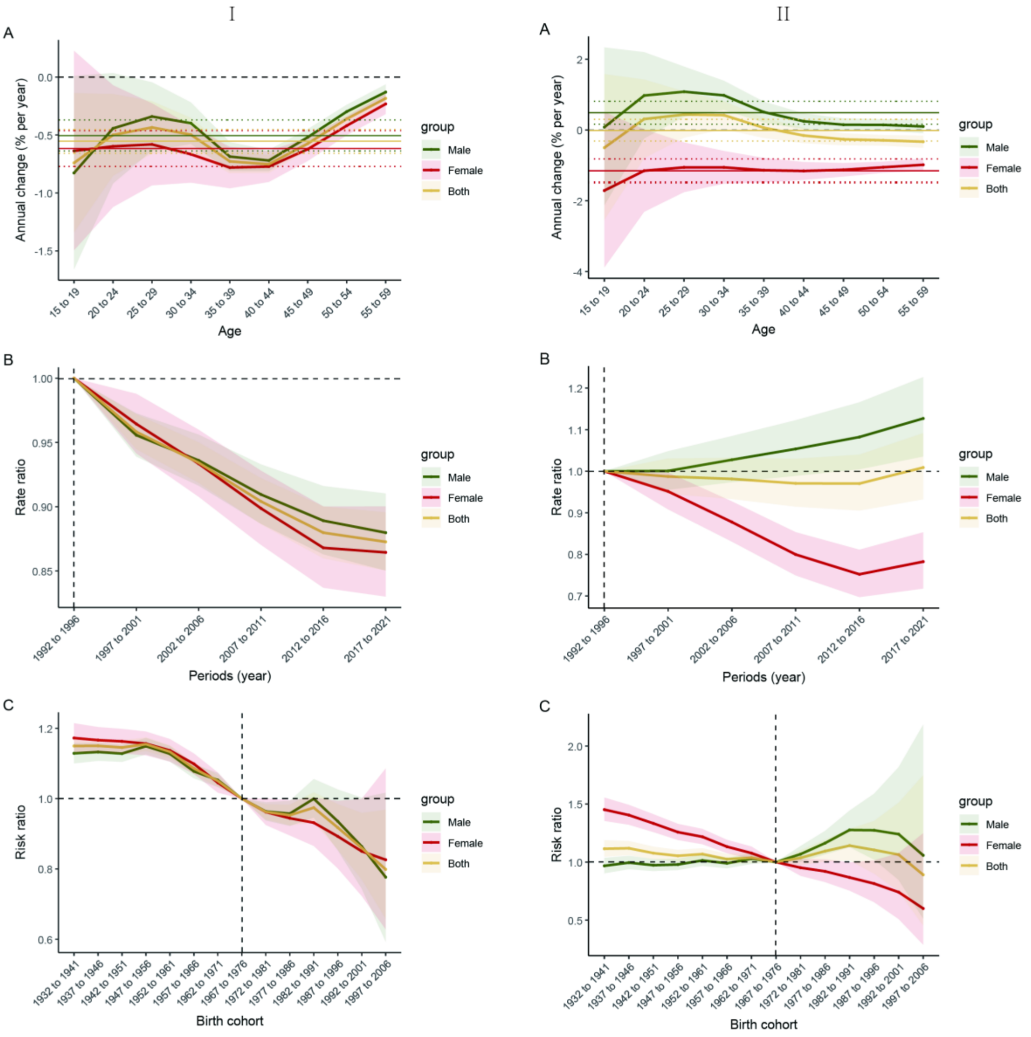

Figure S-3: Age, Period, and Birth Cohort Effects on 15 - 59 - year - old Pancreatic Cancer Mortality.I:Global;II:China.A:Age Cohort; B:Period Cohort; C:Birth Cohort.

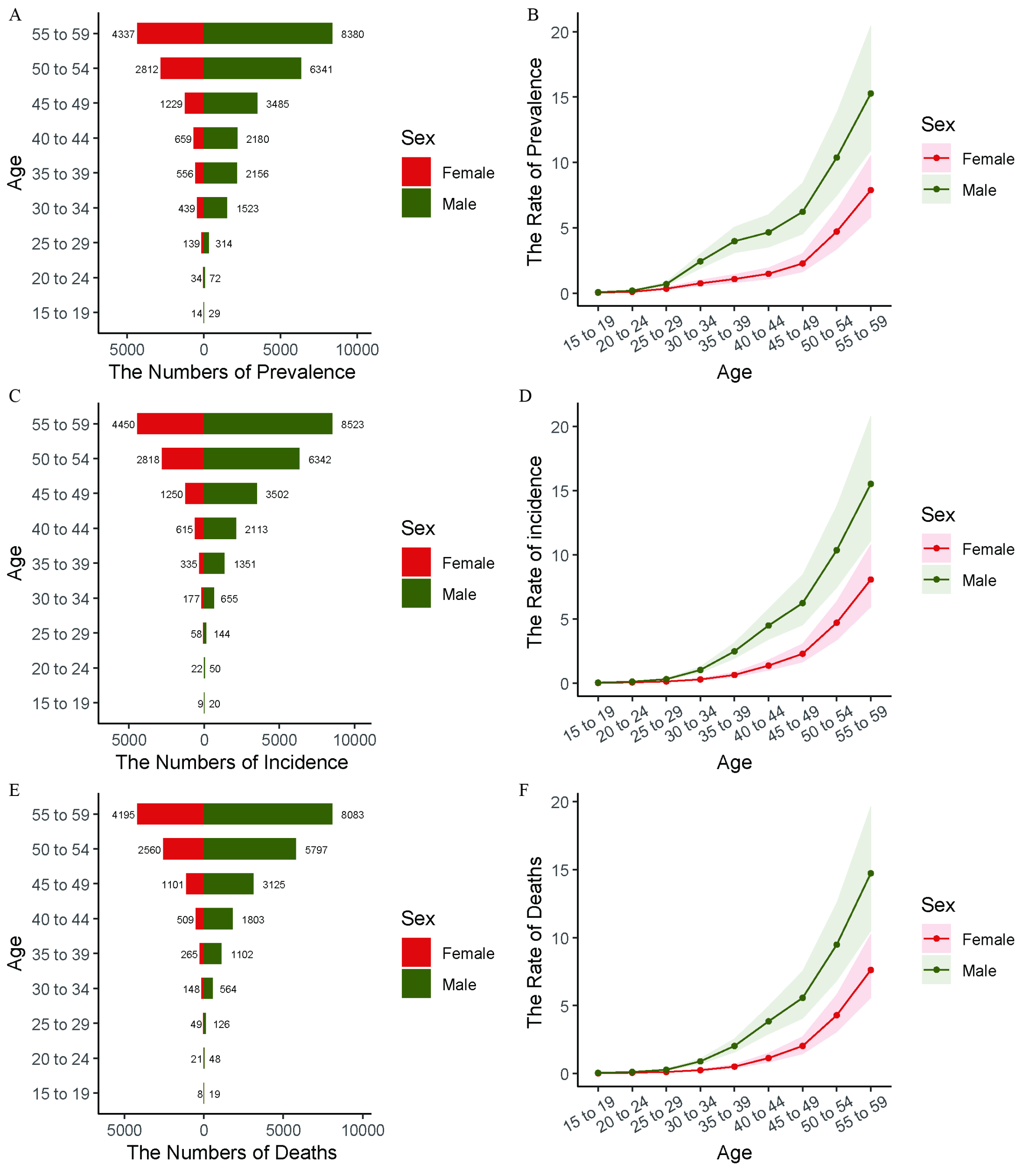

Figure S-4: Pancreatic cancer prevalence, incidence, and mortality among 15–59-year-olds in China, by age and gender. In (**A**,**C**,**E**), bar charts show counts for each age group (red for females, green for males, values labeled above). In (**B**,**D**,**F**), curves show rate trends (red for females, green for males); Women are shown on the left.
